# Vertical Transmission of Bacterial Eye Infections, Angola, 2011–2012

**DOI:** 10.3201/eid2103.140312

**Published:** 2015-03

**Authors:** Mar Justel, Isabel Alexandre, Prudencio Martínez, Iván Sanz, Ana Rodriguez-Fernandez, Itziar Fernandez, Jose Carlos Pastor, Raúl Ortiz de Lejarazu

**Affiliations:** Hospital Clínico Universitario, Valladolid, Spain (M. Justel, A. Rodriguez-Fernandez, J.C. Pastor, R. Ortiz de Lejarazu);; Agostinho Neto University Medical School, Luanda, Angola (I. Alexandre);; Instituto Oftalmologico Nacional, Luanda (I. Alexandre); Redigal, Oviedo, Spain (P. Martinez);; Centro Nacional de Gripe, Valladolid (I. Sanz, R. Ortiz de Lejarazu);; Instituto de Salud Carlos III, Madrid, Spain (I. Fernandez);; Instituto Oftalmobiologia Aplicada, Valladolid (I. Fernandez, J.C. Pastor);; Universidad de Valladolid, Valladolid (J.C. Pastor, R. Ortiz de Lejarazu)

**Keywords:** Chlamydia trachomatis, Neisseria gonorrhoeae, Mycoplasma genitalium, bacterial eye infections, sexually transmitted diseases, molecular diagnostic techniques, vertical infectious disease transmission, bacteria, eye, Angola

## Abstract

To determine transmission rates for neonatal conjunctivitis causative microorganisms in Angola, we analyzed 312 endocervical and 255 conjunctival samples from mothers and newborns, respectively, during 2011–2012. Transmission rates were 50% for *Chlamydia trachomatis* and *Neisseria gonorrhoeae* and 10.5% for *Mycoplasma genitalium*. Possible pathogenic effects of *M. genitalium* in children’s eyes are unknown.

Ophthalmia neonatorum (neonatal conjunctivitis) can be easily prevented with prophylactic administration of topical antimicrobial drugs, as recommended by global guidelines for the management of sexually transmitted infections (*1*). However, in Angola, routine prenatal and prophylactic care to prevent this disease in newborns is lacking. In 2009, a project aimed at developing a national program for ophthalmia neonatorum prophylaxis in all maternity wards of Angola was started through the Spanish Agency for International Cooperation and Development and with the collaboration of the Angolan Ministry of Health. A previous pilot study in Luanda, Angola, showed that clinical cases of acute conjunctivitis among newborns were frequent; ≈12% of infants were born with bilateral acute conjunctivitis (*2*). However, the absence of microbiology laboratory resources at the study site resulted in the inability to determine the causative pathogens. One of the phases of the program was to analyze the usefulness of molecular biology tests to detect 2 of the most common sexually transmitted pathogens associated with transmission from the mother to the eye of the child—*Chlamydia trachomatis* and *Neisseria gonorrhoeae* (*3*)—and an emerging third pathogen (*4*), *Mycoplasma genitalium*. Our aim with this study was to assess the frequency of these 3 infections in a sample of mothers and their newborns in Angola and to determine the rate of vertical transmission.

## The Study

In this prospective, observational study, from December 2011 through February 2012, pregnant women and their newborns were recruited from 2 obstetric clinical wards at the Augusto N’Gangula Hospital and the Health Center of Samba in Angola. Study participation was voluntary. Specific informed consent was obtained from all participants before specimens were collected. Approval of the study protocol for scientific and ethical aspects was obtained from the Ethical Commission of the School of Medicine of the Agostinho Neto University (Luanda, Angola). The study was performed in accordance with the ethical standards of the 1964 Declaration of Helsinki and later amendments. 

Included in the study were mothers who were healthy (with the possible exception of genitourinary disease) and their newborns with a gestational time of 37–40 weeks and a weight of >2.3 kg. Endocervical samples were obtained consecutively from pregnant women who agreed to participate in the study and were collected after removal of postpartum secretions from the endocervical os. Ocular samples were obtained from both eyes of the newborns by vigorous swabbing across the inferior tarsal conjunctiva. Samples from each eye were then pooled for analysis. After sampling, the newborns were prophylactically given 5% povidone iodine eyedrops.

Samples were collected with flocked swabs in Universal Transport Medium (Copan Italia S.p.A., Brescia, Italy), stored at −70°C, and shipped to the Department of Microbiology at the Hospital Clínico Universitario of Valladolid, Valladolid, Spain. DNA extraction was performed according to routine laboratory standards with the GXT DNA/RNA reagents in a GenoXtract extractor (HainLifescience, Nehren, Germany). A multiplex PCR that co-amplified DNA sequences of *C. trachomatis*, *N. gonorrhoeae*, *M. genitalium*, and an internal control was performed by using the Bio-Rad Dx CT/NG/MG assay (Bio-Rad, Hercules, CA, USA) (*5*). The primers and probe for *C. trachomatis* targeted a sequence of the cryptic plasmid located outside the region deleted in the new variant strain of *C. trachomatis* (*6*). For *N. gonorrhoeae*, the target was a sequence in the *pilE* gene that rarely yields false-positive results. For *M. genitalium*, the target was a sequence in the *MgPa* gene. To avoid laboratory or sampling errors, we processed the samples from the mothers and newborns separately and included positive and negative controls in the PCRs. Amplification and detection were performed in a 7500Fast Real-Time PCR system (Life Technologies, Carlsbad, CA, USA), according to the manufacturer’s instructions. Statistical analyses were performed by using R Statistical Software (Foundation for Statistical Computing, Vienna, Austria).

A total of 567 samples were analyzed (Table 1). Results of detection by multiplex PCR were considered for prevalence estimates of the 3 infections (Table 2). Transmission rates were 50% for *C. trachomatis* and *N. gonorrhoeae* and 10.5% for *M. genitalium*. For 7 infected mothers (2 *C. trachomatis*–infected and 5 *M. genitalium*–infected), no samples could be obtained from their newborns.

**Table 1:** Samples collected for analysis of vertical transmission of eye infections, Angola, 2011–2012

Samples	Augusto N’Gangula	Health Center of Samba	Total
Endocervical	169	143	312
Conjunctival*	130	125	255
Total	299	268	567
*For reasons other than exclusion criteria, samples from 57 newborns were not obtained.			

**Table 2:** Prevalence of microorganisms among mothers and newborns from Augusto N´Gangula Hospital and Health Center of Samba in Luanda, Angola, 2011–2012

Microorganism, sample type	No. positive/no. collected	Prevalence, % (95% CI)
*Chlamydia trachomatis*		
Endocervical	8/312	2.6 (1.3–4.9)
Conjunctival	4/255	1.6 (0.6–3.9)
Total	12/567	2.1 (1.2–3.6)
*Neisseria gonorrhoeae*		
Endocervical	2/312	0.6 (0.2–2.3)
Conjunctival	1/255	0.4 (<0.1–2.2)
Total	3/567	0.5 (0.2–1.5)
*Mycoplasma genitalium*		
Endocervical	19/312	6.1 (3.9–9.3)
Conjunctival	2/255	0.8 (0.2–2.8)
Total	21/567	3.7 (2.4–5.6)
All 3 microorganisms		
Endocervical	28/312*	9.0 (6.3–12.6)
Conjunctival	7/255	2.7 (1.3–5.5)
Total	35/567*	6.2 (4.5–8.5)

*One mother was co-infected with *C. trachomatis* and *M. genitalium*.

## Conclusions

The microorganism most frequently found among mothers was *M. genitalium*, and the microorganism most frequently found among newborns was *C. trachomatis*. Despite the association of *M. genitalium* with sexually transmitted infections in men and women (*4*), we are aware of only 1 other case of conjunctivitis associated with *M. genitalium* (*7*).

Only 1 mother was co-infected by 2 microorganisms, *C. trachomatis* and *M. genitalium*, consistent with the low rate of this co-infection found in other studies (*8*). This mother gave birth to 1 of the 4 *C. trachomatis*–infected newborns. Not answered by this study is the hypothetical role of *M. genitalium* as a co-factor for transmission of other major sexually transmitted pathogens. *M. genitalium* has also been studied as a possible contributor to the pathogenesis of trachoma in a trachoma-endemic area of Tanzania, but no evidence was found regarding its contribution (*9*). Our findings regarding *M. genitalium* infections in this sample need further study because the relatively small number of infected mothers and children can be a confounding factor and because the real prevalence of *M. genitalium* needs to be assessed by larger studies.

The frequency of *C. trachomatis* infection among the study sample (mothers and newborns), 2.1%, was lower than that found in previous studies in different populations in Africa (*10*). The frequency of *N. gonorrhoeae* infection among the study sample, 0.5%, was also lower than that found in other African countries, such as the Central African Republic (3.1%) and South Africa (7.8%) (*11*). The frequency of *M. genitalium* infection among the study mothers, 6.1%, was slightly higher than that found in other studies of asymptomatic women (*8*).

Rates of transmission from the mother to the eye of the child differed markedly for each of the 3 microorganisms studied. The rate of *M. genitalium* transmission was much lower than that for *C. trachomatis* and *N. gonorrhoeae*. Vertical transmission of *M. genitalium* is uncommon; we are aware of only 1 reported case (*12*). Although *M. genitalium* is among the most prevalent pathogens in this sample of mothers in Africa, vertical transmission of this microorganism was less frequent than that of other classical sexually transmitted pathogens like *C. trachomatis* and *N. gonorrhoeae*. However, high prevalence in any maternal population, even with a relative low rate of transmission, could lead to a large number of neonatal infections. 

All infected children were from mothers who were also infected. It can be assumed that, as with HIV infection, detection of any of the assayed microorganisms in conjunctival samples of newborns predicts diagnosis in their mothers (*13*). This so-called mirror effect is clinically useful in view of cultural behaviors that would complicate detection of sexually transmitted pathogens in adult women, especially in geographic settings of Muslim practices (*14*).

The major limitation of this study was the difficulty of correctly preserving samples in the hot and humid environment of Angola. To prevent DNA denaturation, samples were frozen immediately after collection and shipped as soon as possible. Unfortunately, those precautions precluded the culturing of samples, so PCR results could not be compared with culture results. Another disadvantage was the lack of more precise clinical information from participants because of the absence of antenatal care for most**.**

Our study findings indicate that new molecular techniques will help microbiological diagnosis of neonatal conjunctivitis in Africa. They also show the need for a national program for neonatal conjunctivitis prophylaxis in Angola, taking into account the most frequent causative microorganism. 
